# Neoadjuvant Programmed Cell Death Protein 1 Blockade Combined With Stereotactic Body Radiation Therapy for Stage III(N2) Non-Small Cell Lung Cancer: A Case Series

**DOI:** 10.3389/fonc.2022.779251

**Published:** 2022-03-07

**Authors:** Zhen Wang, Yong Qiang, Qin Shen, Xi-Xu Zhu, Yong Song

**Affiliations:** ^1^Department of Radiation Oncology, Jinling Hospital: East Region Military Command General Hospital, Nanjing, China; ^2^Department of Cardiothoracic Surgery, Jinling Hospital: East Region Military Command General Hospital, Nanjing, China; ^3^Department of Pathology, Jinling Hospital: East Region Military Command General Hospital, Nanjing, China; ^4^Department of Respiratory and Critical Medicine, Jinling Hospital: Jinling Hospital, Medical School of Nanjing University, Nanjing, China

**Keywords:** Neoadjuvant, radiation therapy, programmed cell death protein 1(PD-1), non-small cell lung carcinoma, stereotactic body radiotherapy (SBRT)

## Abstract

The addition of radiotherapy in neoadjuvant chemotherapy did not improve event-free or overall survival in resectable non-small cell lung carcinoma (NSCLC). Neoadjuvant immunotherapy produced major pathologic response(MPR) rate of up to 45%. The potential synergy between radiotherapy and immunotherapy has been described in several studies. We reported outcomes of three cases of stage III/N2 NSCLC treated with neoadjuvant immunotherapy and stereotactic body radiation therapy (SBRT) in a single center. This explanatory trial included treatment-naive patients with stage III resectable NSCLC who received two doses of the programmed cell death protein 1 (PD-1) inhibitor toripalimab after 1 week of receiving SBRT for lung lesions. Thereafter, surgery was planned 4–6 weeks after the second dose. The primary endpoints were safety and feasibility, while the secondary endpoint was the pathologic response rate. Toripalimab combined with SBRT as a neoadjuvant treatment had well-tolerable side effects and did not lead to a delay in surgery. Among the included patients, one achieved pathologic complete response (PCR), one achieved MPR, and one with 20% residual tumor did not achieve MPR. There were few side effects of toripalimab combined with SBRT as a neoadjuvant treatment, and the treatment did not cause a delay in surgery. This study preliminarily explored the outcomes of a new neoadjuvant treatment.

## Introduction

Effective treatments are needed for patients with stage III NSCLC, which has a 5-year survival rate ranging from 13% to 36%, with most patients having postsurgical tumor relapse (1). Reports on long-term outcomes of patients with stage III NSCLC beyond 5 years who have received multimodal treatment including surgery are scarce. Perioperative neoadjuvant platinum-based chemotherapy is associated with a survival rate that is only 5 percentage points higher than that with surgery alone, with rates of toxic effects of grade 3 or higher of more than 60% (2). The addition of radiotherapy in neoadjuvant chemotherapy did not improve event-free or overall survival in stage III/N2 non-small cell lung carcinoma (NSCLC) (3, 4). Hence, neoadjuvant treatment regimens have remained a research focus for clinicians.

At present, antibodies that block PD-1 immune suppression pathway provide an important option for treating patients with lung cancer. For patients stage III unresectable NSCLC that had not progressed after chemoradiotherapy, these antibodies can activate antitumor immunity, causing tumors to shrink, thereby improving survival (5, 6). In the neoadjuvant setting, Forde et al. first reported the safety and efficacy of neoadjuvant nivolumab in a study on 22 patients with early NSCLC along with a postoperative MPR rate of 45%, which is higher than the rate of conventional neoadjuvant chemotherapy (approximately 20%–30%) (7).

Herein we reported the outcomes of three patients with advanced stage III NSCLC treated with neoadjuvant immunotherapy combined with SBRT and surgery at our institution. The patient and tumor characteristics are detailed in **Table 1** and **Table S1**. Patients were administered SBRT on the first week, followed by two doses of intravenous toripalimab (an anti-PD-1 antibody) (240 mg) every 3 weeks. Surgery was planned approximately 4-6weeks after the second dose, followed by maintenance toripalimab monotherapy for one year. All patients provided written informed consent. The primary endpoints were safety and feasibility, while the secondary endpoint was the pathologic response rate.

**Table 1:** Timeline with relevant data from the episode of care of three patients.

Patient	Time from SBRT to first immunotherapy	Time from last immunotherapy to surgery	Pathologic type	Genetic Testing	TMB	PD-L1 (22C3)	Radiologic evaluation	Surgery procedure	Pathologic evaluation	Toxic side effects	Follow UP
Patient 1	2 weeks	4 weeks	Adenocarcinoma	EGFR (-) ALK (−) KRAS (+) TP53 (+)	18.46 Muts/Mb	70%	PR	Thoracotomy surgery R0	CPR	Grade 2 RF Grade 2 pruritus	12months
Patient 2	2 weeks	6 weeks	Adenocarcinoma	EGFR (−) ALK (−)	–	0%	SD (shrunk by 29%)	Thoracoscopy R0	No-MPR (20% residue)	No	11months
Patient 3	2 weeks	9 weeks	Adenocarcinoma	EGFR ALK (−) ERBB2 (+)	9.3Muts/Mb	10%	PR	Thoracotomy surgery R0	MPR	Postoperative chest pain	12months

CPR, Complete Pathologic Response; MPR, Major pathological response; RF, radiation fibrosis.

## Case Description

**Figure 1:**
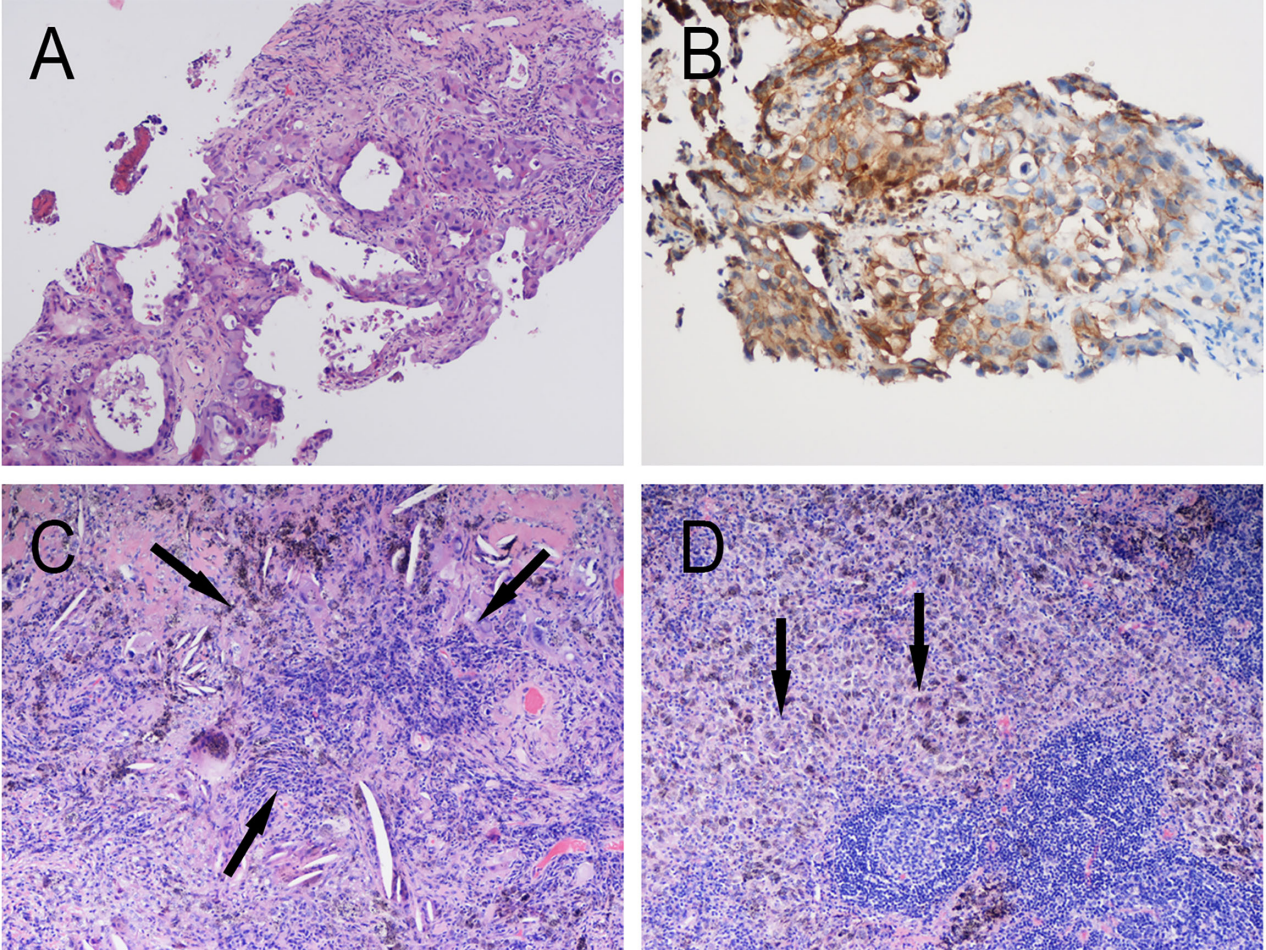
Pathology of Patient 1. **(A)** Hematoxylin and eosin (HE) staining of pretreatment tumor biopsy shows invasive adenocarcinoma (moderate–low differentiation, acinar-solid subtype) and **(B)** PD-L1 high expression, approximately 80% TPS. Postoperative pathology: Extensive fibrosis and necrosis with no residual viable cancer cells in the left upper lung lesion **(C)** and hilar lymph nodes **(D)**.

**Figure 2:**
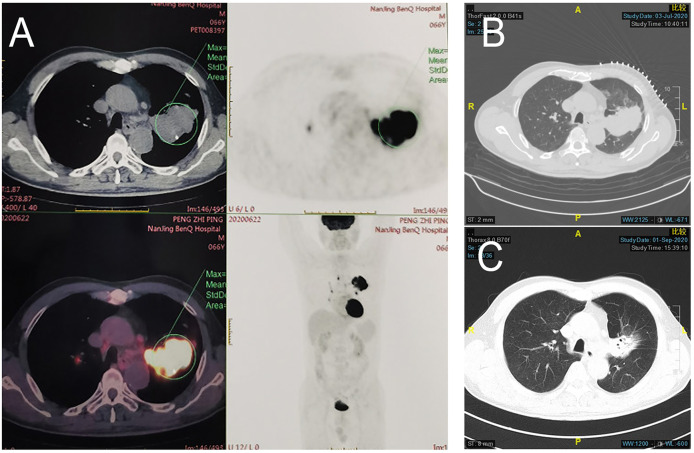
Radiologic examination of Patient 1. **(A)** Baseline PET/CT showed a mass near the pulmonary hilum of the left upper lung lobe that was approximately 6.7 × 4.2 cm in size with an SUV_max_ of 17.7. Multiple enlarged lymph nodes can be observed at the left lung hilum and mediastinal stations 4 and 5, with an SUV_max_ of 11.1. CT scans of the chest before **(B)** and after **(C)** treatment showed shrinking of left upper lung lesions, with radiologic evaluation showing PR (RECIST 1.1).

**Patient 1:** In June 2020, a 68-year-old man with complaints of cough and expectoration of bloody sputum was admitted to Jinling Hospital. He had no significant medical history besides smoking one cigarette pack per day for 50 years. Histologic analysis of CT-guided percutaneous lung puncture biopsies of the primary lesion showed poorly differentiated adenocarcinoma in the lung (**Figure 1A**). Immunohistochemistry (IHC) showed TTF1 (3+) expression, NapsinA (1+)-positive (weak) staining, P40-negative staining, and a KI67 index of 20%. In addition, IHC showed PD-1 ligand 1 (PD-L1) expression in 80% of the cancer cells (**Figure 1B**), without ALK or ROS-1 expression. Next-generation sequencing (NGS) did not reveal any TKI-targetable mutations and high TMB (18.46 Muts/Mb). TNM classification (eighth edition) based on fluorideoxyglucose positron-emission tomography combined with CT (FDG-PET/CT) (**Figure 2A**) and contrast-enhanced brain magnetic resonance imaging (MRI) was cT3 cN2b cM0, consistent with stage cIIIB disease. T3 was based on tumor size (6.7cm), whereas N2 was based on a hypermetabolic enlargement of mediastinal lymph nodes in stations 4L and 5, without cyto/histologic confirmation.

The patient was administered SBRT on the first week, followed by two doses of toripalimab (240 mg) every 3 weeks. SBRT was administered at a total dose of 50 Gy over 5 days. The dose equivalence was estimated using a linear quadratic model and considered by assuming *α/β *= 10 Gy for the tumor. Biologically equivalent dose (BED) was 100 Gy.

Computed tomographic (CT) scans after two cycles of toripalimab showed tumor partial response(PR), with a significant decrease in the left lung mass (**Figures 2B, C**). Moreover, brain MRI excluded cerebral metastases. Thoracotomy surgery, left upper lobectomy with venous graft bypass between the left pulmonary artery and inferior lobar artery, and mediastinal lymph node dissection were performed in September 2020, 4 weeks after the second toripalimab dose. Histologic analysis of the resected lung and lymph nodes showed an absence of viable tumor cells in addition to pulmonary necrosis and fibrosis (**Figures 1C, D**). Hence, the pathologic TNM classification (eighth edition) was ypT0 ypN0 (R0). Toripalimab was continued after the surgery. In March 2021, 6 months after surgical resection, the patient was still in complete remission but suffered from Grade 2 radiation fibrosis (RF) and Grade 2 immune-related pruritus.

**Figure 3:**
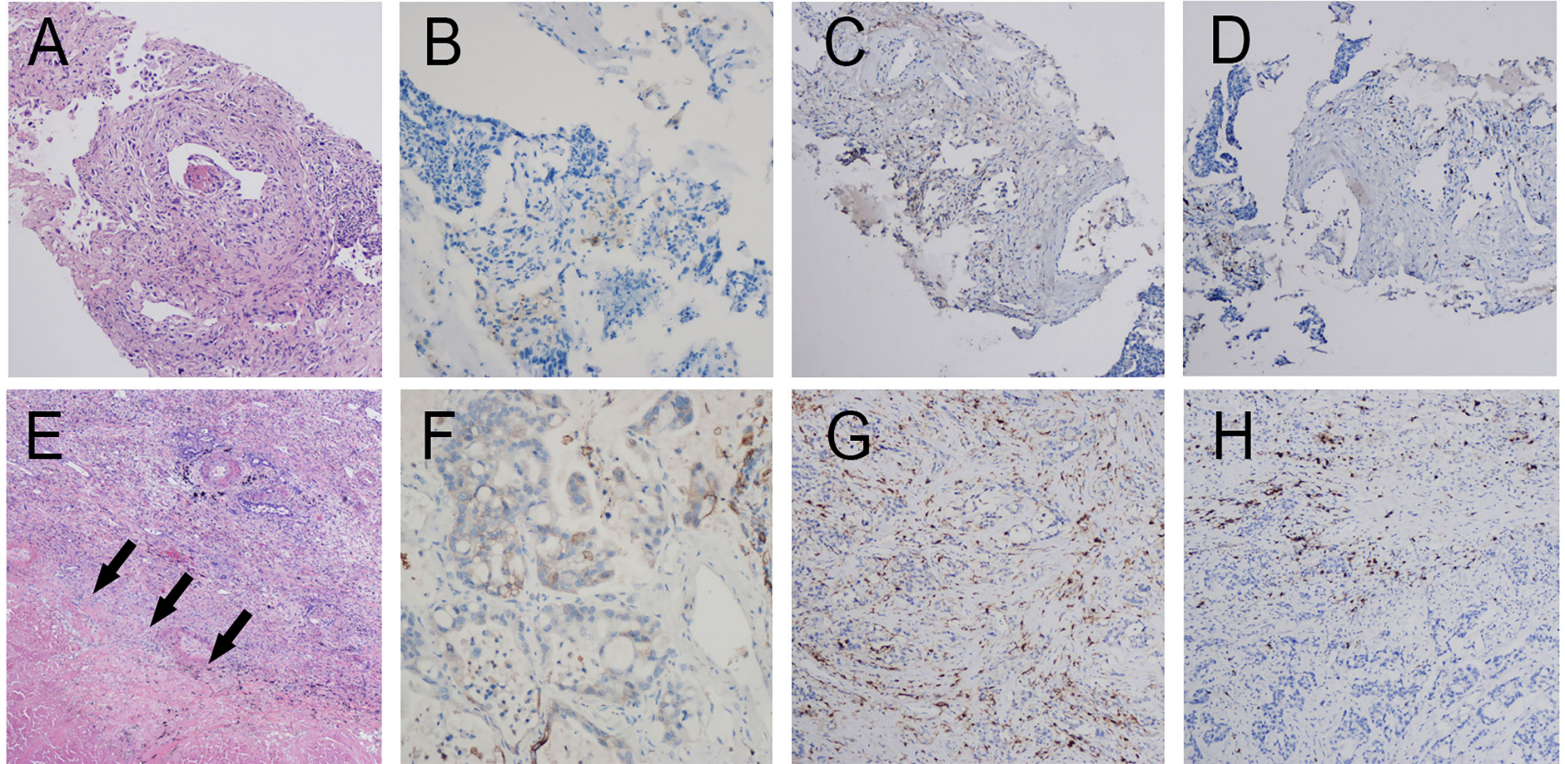
Pathology of patient 2. Pathology of pretreatment tumor biopsy shows poorly differentiated adenocarcinoma **(A)** accompanied by **(B)** PD-L1 negative (0% TPS) as well as few infiltrating lymphocytes. (CD4 cells accounting for approximately 5% in the hotspot region **(C)**. CD8 cells accounting for approximately 10% in the hotspot region **(D)**. Postoperative pathology shows poorly differentiated invasive adenocarcinoma **(E, F)** PD-L1 negative (1% TPS). Large patches of necrosis, histiocytic reactions, and cholesterol crystal deposition were noted in addition to lymphocyte infiltration (CD4 T cells **(G)** and CD8 T cells **(H)** accounting for approximately 10% and 15% in the hotspot region, respectively).

**Patient 2:** In June 2020, a 63-year-old man with complaints of left-sided chest pain was admitted to Jinling Hospital. He had no significant medical history besides smoking one cigarette pack per day for 40 years. Histologic analysis of CT-guided percutaneous lung puncture biopsies of the primary lesion showed poorly differentiated adenocarcinoma in the lung (**Figure 3A**). IHC showed TTF1 expression (2+), NapsinA (lesion 2+), CKpan (3+)-positive staining, and a KI67 index of 30%. Moreover, IHC showed PD-L1 expression in 0% of the cancer cells (**Figure 3B**) and small numbers of CD4 and CD8 T cells (**Figures 3C, D**). EGFR\ROS1\HER2\NRAS\BRAF\PIK3CA\KRAS and ALK tests were negative. TNM classification (eighth edition) based on contrast-enhanced CT and brain MRI was cT3 cN2a2 cM0, consistent with stage cIIIB disease. CT showed two adjacent lesions in the left lower lobe, with sizes of 4.0cm and 2.7cm, respectively. The short diameter of the subcarinal lymph node was 1.0cm and the biopsy by endobronchial ultrasound-guided transbronchial needle aspiration biopsy showed a small number of tumor cells. This patient was also administered SBRT at the left lower lung lesion(50Gy/5fractions) and two cycles of toripalimab.

**Figure 4:**
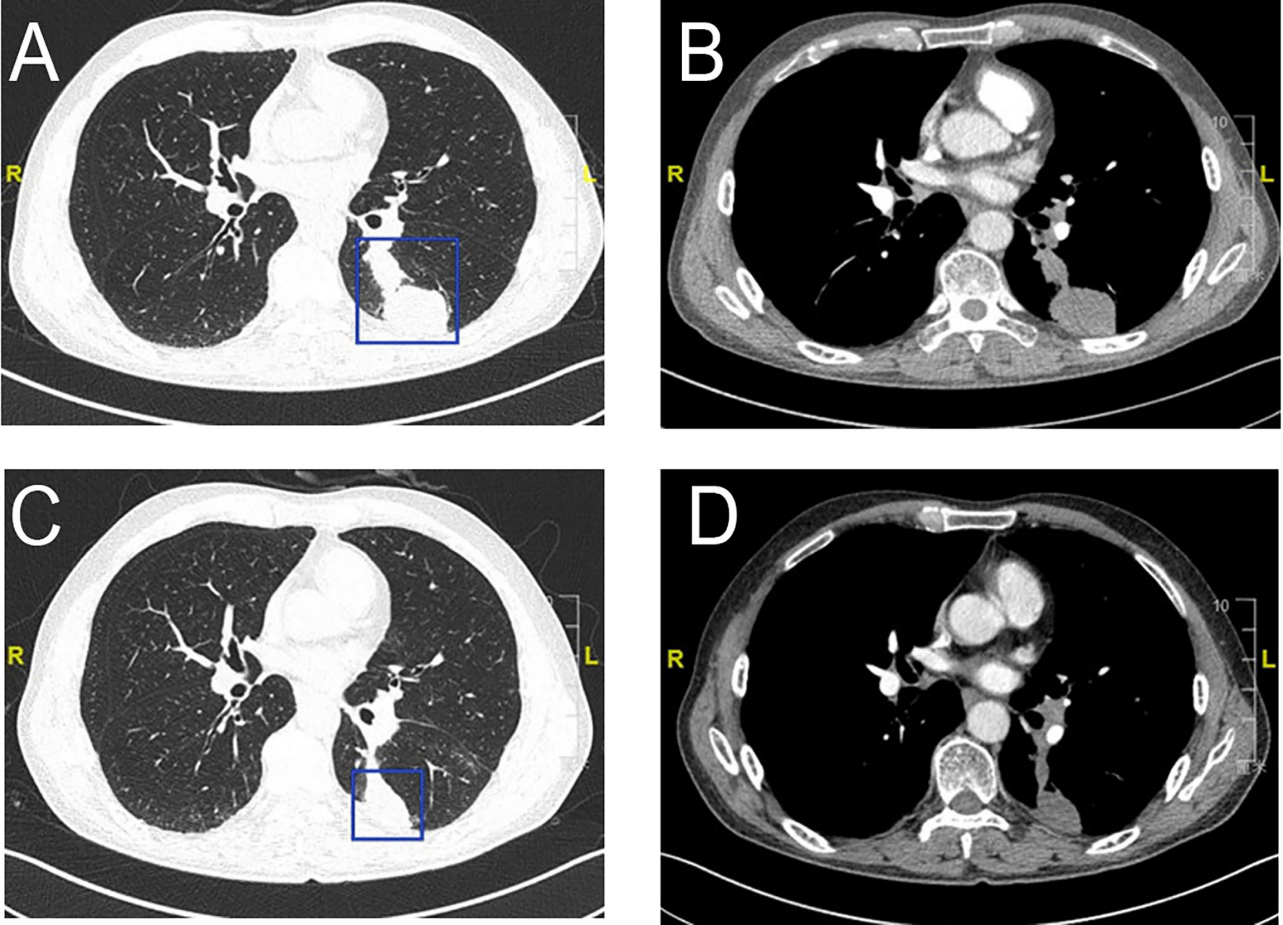
Radiologic examination of Patient 2. CT scans of the chest shows two adjacent lesions in the left lower lobe of the lung **(A, B)**. A scan performed before surgery shows 29% shrinkage **(C, D)**.

CT scans after two cycles of toripalimab showed a minor response (left lower lung lesion decreased by 29%) (**Figure 4**). Brain MRI excluded cerebral metastases. Thoracoscopic surgery, left upper lobectomy with venous graft bypass between the left pulmonary artery and inferior lobar artery, and mediastinal lymph node dissection were performed in September 2020, 4 weeks after the second dose of toripalimab.

Invasive adenocarcinoma was observed in the postoperative lung and lymph nodes specimens accompanied by large numbers of necrotic cells (**Figure 3E**). Comprehensive evaluation of all lesions showed that tumor residues accounted for 20%. Although Major pathological response(MPR) was not achieved, an increase in the number of CD4 and CD8 T cells was observed (**Figures 3G, H**). PD-L1 expression levels are also increased (**Figure 3F**). Therefore, the pathologic TNM classification (eighth edition) was ypT3 ypN2 (R0). Toripalimab was continued after the surgery. In March 2021, 5 months after surgical resection, the patient underwent 6 cycles of immunotherapy as maintenance treatment, with no adverse reactions observed.

**Figure 5:**
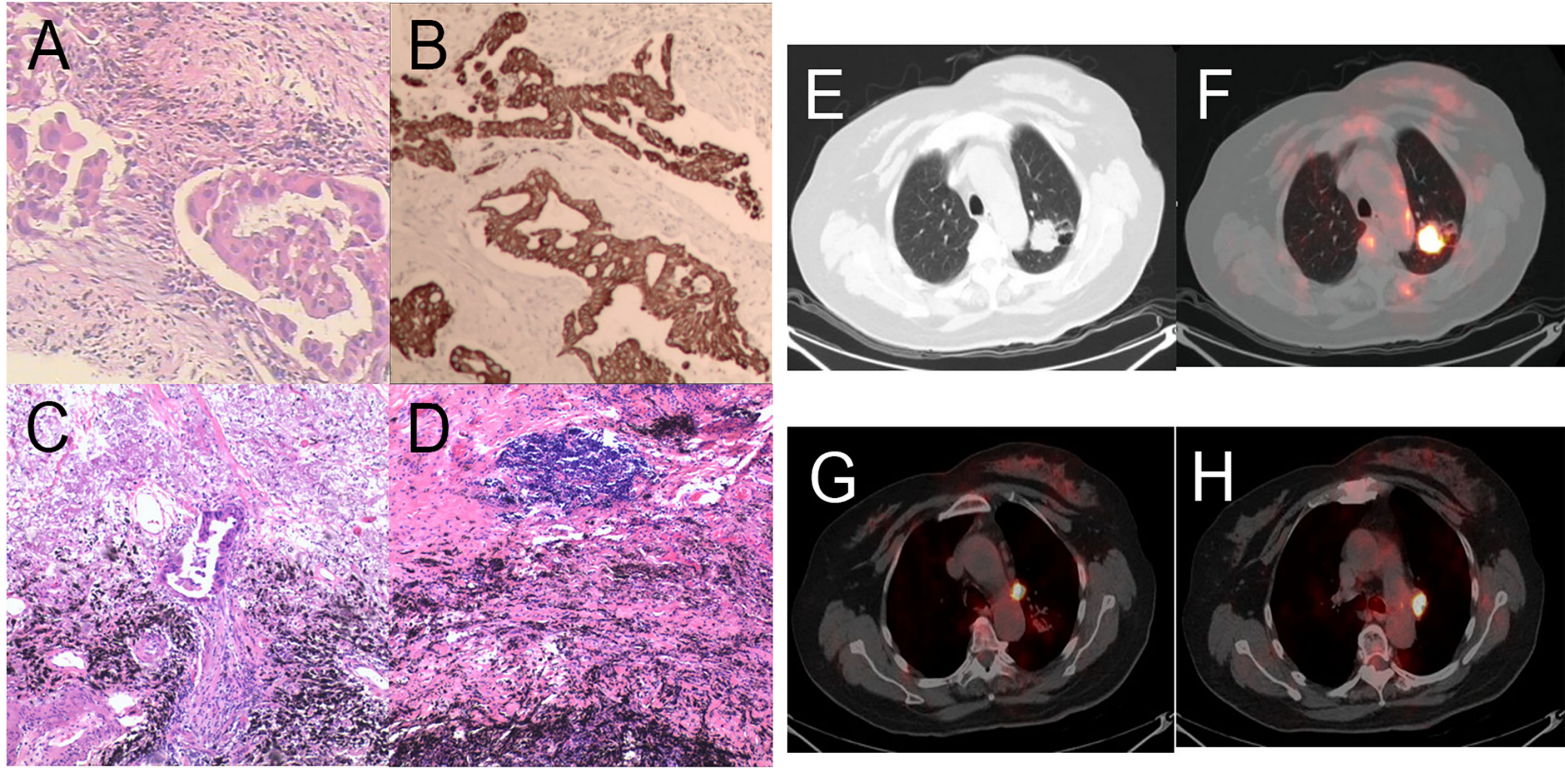
Pathology and PET/CT of Patient 3. **(A)** Hematoxylin and eosin (HE) staining of pretreatment tumor biopsy shows invasive adenocarcinoma and **(B)** PD-L1 high expression, approximately 10% TPS. There was more than 90% tumor regression **(C)** in the left upper lung lobe, no residual viable cancer cells in hilar lymph nodes **(D)** in the post-treatment specimens. **(E, F)** A round mass (approximately 32 × 27 mm in size and SUV_max_ of 9.35) can be observed at the posterior segment of the left upper lung lobe. **(G, H)** Multiple enlarged lymph nodes can be seen at the left lung hilum and mediastinal station 6 with an SUV_max_ was 8.1 and 8.8, respectively.

**Patient 3:** In May 2020, a 66-year-old woman suffering from cerebral infarction was admitted to Jinling Hospital. She had no history of smoking but had a medical history of hypertension and diabetes and was on long-term oral anticoagulants. Histologic analysis of CT-guided percutaneous lung puncture biopsies of the primary lesion showed adenocarcinoma in the lung (**Figure 5A**). IHC showed TTF1 expression (+), NapsinA (−), and a KI67 index of 10%. Moreover, IHC showed PD-L1 expression in 10% of cancer cells (**Figure 5B**). NGS did not reveal any TKI-targetable mutation; however, ERBB2 was positive. TNM classification (eighth edition) based on FDG-PET/CT and contrast-enhanced brain MRI was cT2a cN2a2 cM0, consistent with stage cIIIA disease. The size of lesion in the right upper lobes was 32mm and the SUVmax-FDG was 9.35. The SUVmax of hilum and mediastinum region 6 (para-aortic arch) metastatic lymph nodes was 8.1 and 8.8, respectively (**Figures 5E–H**). Surgery could not be performed because of acute cerebral infarction. Therefore, the patient chose to first undergo neoadjuvant treatment, followed by surgery depending on her condition. A total of 50 Gy/5 fractions SBRT was administered to the left lower lung lesion along with two cycles of toripalimab.

CT scans after two cycles of toripalimab showed a minor response, with a 20% decrease in the left lung mass. Brain MRI excluded cerebral metastases. However, new-onset cerebral infarction occurred again. Therefore, cerebral infarction was first treated briefly. Thereafter, Toripalimab was administered on September 1 and 24, 2020. PET/CT before surgery was shown in **Figure S1**. Left upper lobectomy and lymphadenectomy were performed on December 01, 2020. Postoperative pathology showed mild glandular dysplasia in the lesion, and invasive adenocarcinoma was considered (**Figure 5C**). Interstitial hyperplasia, degeneration/necrosis, and infiltration by large numbers of chronic inflammatory cells and histiocytes were observed. The dissected mediastinal and hilar lymph nodes were all negative (**Figure 5D**). Residual tumor was 1% in the comprehensive evaluation, and MPR was achieved. Therefore, pathologic TNM classification (eighth edition) was ypT3 ypN0 (R0). Chest pain appeared after surgery (pain score: 5 points) and gradually improved 1 month after surgery. The patient is currently on maintenance immunotherapy for four cycle, and Grade 3 or above adverse reactions were not observed.

## Discussion

The present study showed that neoadjuvant SBRT combined with immunotherapy was safe in three patients with stage III NSCLC. There was no delay in surgery occurred due to toxic side effects. In addition, severe perioperative complications were absent, and Grade 3 or above pneumonitis did not occur. Among the three patients, one achieved pathologic complete response and one achieved MPR. Our results were similar to other studies on neoadjuvant immunotherapy (7, 8). The present cases showed that there is a difference between radiologic and pathologic response, and persistent tumor masses do not represent residual active cancer.

In the chemotherapy era, several studies have shown that the addition of radiotherapy in neoadjuvant chemotherapy did not improve PCR and event-free or overall survival in stage III/N2 NSCLC. The possible reason is that the conventional radiotherapy dose is insufficient, which prolongs the waiting time for surgery (3, 4). SBRT is a precise radiotherapy model that can significantly increase the radiation dose but reduce damage to normal tissues. SBRT has shown good efficacy in treating lung cancer and inducing antitumor immune responses better than conventional radiotherapy (9–11). Neoadjuvant SBRT was confirmed well tolerated, safe, and the pathologic complete response rate after SABR for early-stage NSCLC was 60% (12). But for stage III(N2) NSCLC, a combination of more effective systemic therapy is required. The appearance of immune checkpoint inhibitors brings new hope for NSCLC. Compared to neoadjuvant chemotherapy, neoadjuvant anti-PD-1 or anti-PD-L1 monotherapy achieved a higher MPR rate in early-stage NSCLC (7, 13, 14) Radiotherapy has shown enhance immune response through multiple mechanisms, including induction of immunogenic cell death with release of neoantigens, upregulating MHC-I expression, increasing tumor-infiltrating immune cells (15, 16). A randomised phase 2 trial of neoadjuvant durvalumab with or without SBRT in patients with stage I-III NSCLC have reported durvalumab plus SBRT(8GyX3 fractions) associated with a high MPR(53.3% vs 6.7%; p<0.0001) (17). One difference to the present study is that, the radiation dose of 50Gy/5fractions is equivalent to a BED of 100Gy, a substantially higher than the 8Gy/3fractions(BED 43.2Gy). Although no grade ≥3 toxicities occurred, it is worth considering whether such a high radical dose is required.

The irradiation fields of standard thoracic radiation for unresectable locally advanced NSCLC include lung lesions and tumor-associated draining lymph nodes (DLN). In contrast to this, SBRT was performed on lung lesions in this study, however, tumor-associated lymph nodes were not irradiated. The main considerations are as follows:1) DLN is a major site where dendritic cells activate antigen-specific CD8 cells (18). 2) High-dose irradiation of lung lesions and metastatic lymph nodes did not bring survival benefits. Higher doses of radiation to the immune system were associated with tumor progression and death following the definitive treatment of stage III NSCLC (19, 20). 3) A preclinical study show that irradiation of the DLN restrains adaptive immune responses through altered chemokine expression and CD8+ T-cell trafficking. Exclusion of the DLN from the RT target volume should be considered when combining SBRT with immune therapy (21). 4) The out-of-field effect was increased from 10% in conventional radiotherapy to 38% in SBRT combined with immunotherapy (22). SBRT performed on lung lesions was not only decreased surgery difficulty, but also reduced damage to lymph nodes caused by irradiation. Of the three patients in this study, lymph node metastases were confirmed by PET-CT before neoadjuvant treatment in Patients 1 and 3. After neoadjuvant treatment, lymph node specimens were collected during surgery, in which cancer cells were unobserved. This result further demonstrates that good control can be achieved even if lymph node metastases are not irradiated and may be due to abscopal effects caused by immunotherapy and SBRT.

Compared with PD-L1 positive patients, SBRT combined with ICI achieves better survival in PD-L1 negative patients. Theelen et al. published a phase II study in which SBRT was administered to a single tumor lesion in patients with advanced NSCLC, followed by pembrolizumab. Subgroup analysis suggested that patients with PD-L1-negative tumors had a significantly longer PFS and OS, indicating that radiation may alter the tumor microenvironment of PD-L1-negative tumors, thereby facilitating the effects of pembrolizumab (23). Of the three patients in this study, Patient 2 was PD-L1 negative prior to treatment. Comparison between postoperative samples and fine-needle aspiration samples found that CD4 and CD8+ T cell counts increased. Although PD-L1 expression was 80% in Patient 1, there was no increase in CD4 and CD8+ T cells when preoperative and postoperative specimens were compared. This requires further evaluation in the future.

We studied neoadjuvant immunotherapy combined with SBRT in only three patients, and the follow-up period was short. A larger study is required to confirm the safety and long-term efficacy of this treatment protocol. At present, prospective studies evaluating neoadjuvant immunotherapy combined with radiotherapy are ongoing, and the results are pending (NCT03237377, NCT0290495, NCT03217071, and NCT02904954). Our center has also registered a similar study (ChiCTR2000029277). Long-term follow-up is required for these studies to determine the safety, long-term efficacy, and irradiation area and dose for neoadjuvant immunotherapy combined with radiotherapy.

## Conclusion

There were few side effects of toripalimab combined with SBRT as a neoadjuvant treatment, and the treatment did not cause a delay in surgery. This study preliminarily explored the outcomes of a new neoadjuvant treatment.

## Data Availability Statement

The original contributions presented in the study are included in the article/**Supplementary Material**. Further inquiries can be directed to the corresponding author.

## Ethics Statement

The studies involving human participants were reviewed and approved by The Ethics Committee of Jinling Hospital 2019NZKY-025-01. The patients/participants provided their written informed consent to participate in this study. Written informed consent was obtained from the individual(s) for the publication of any potentially identifiable images or data included in this article.

## Author Contributions

ZW and YS suggested and completed the writing of the article. YQ, QS, and X-XZ collected patient data and completed the language polishing of the article. All authors contributed to the article and approved the submitted version.

## Funding

This work was supported by grants from Key Project of Jiangsu Social Development (No. BE2019719).

## Conflict of Interest

The authors declare that the research was conducted in the absence of any commercial or financial relationships that could be construed as a potential conflict of interest.

## Publisher’s Note

All claims expressed in this article are solely those of the authors and do not necessarily represent those of their affiliated organizations, or those of the publisher, the editors and the reviewers. Any product that may be evaluated in this article, or claim that may be made by its manufacturer, is not guaranteed or endorsed by the publisher.
