# Supplementary material for: Neoadjuvant Programmed Cell Death Protein 1 Blockade Combined With Stereotactic Body Radiation Therapy for Stage III(N2) Non-Small Cell Lung Cancer: A Case Series
Source: Front Oncol. 2022 Mar 7;12:779251. doi: 10.3389/fonc.2022.779251 (PMC8936067; doi:10.3389/fonc.2022.779251)
Supplement: Supplementary file 1 [file DataSheet_1.docx]

**
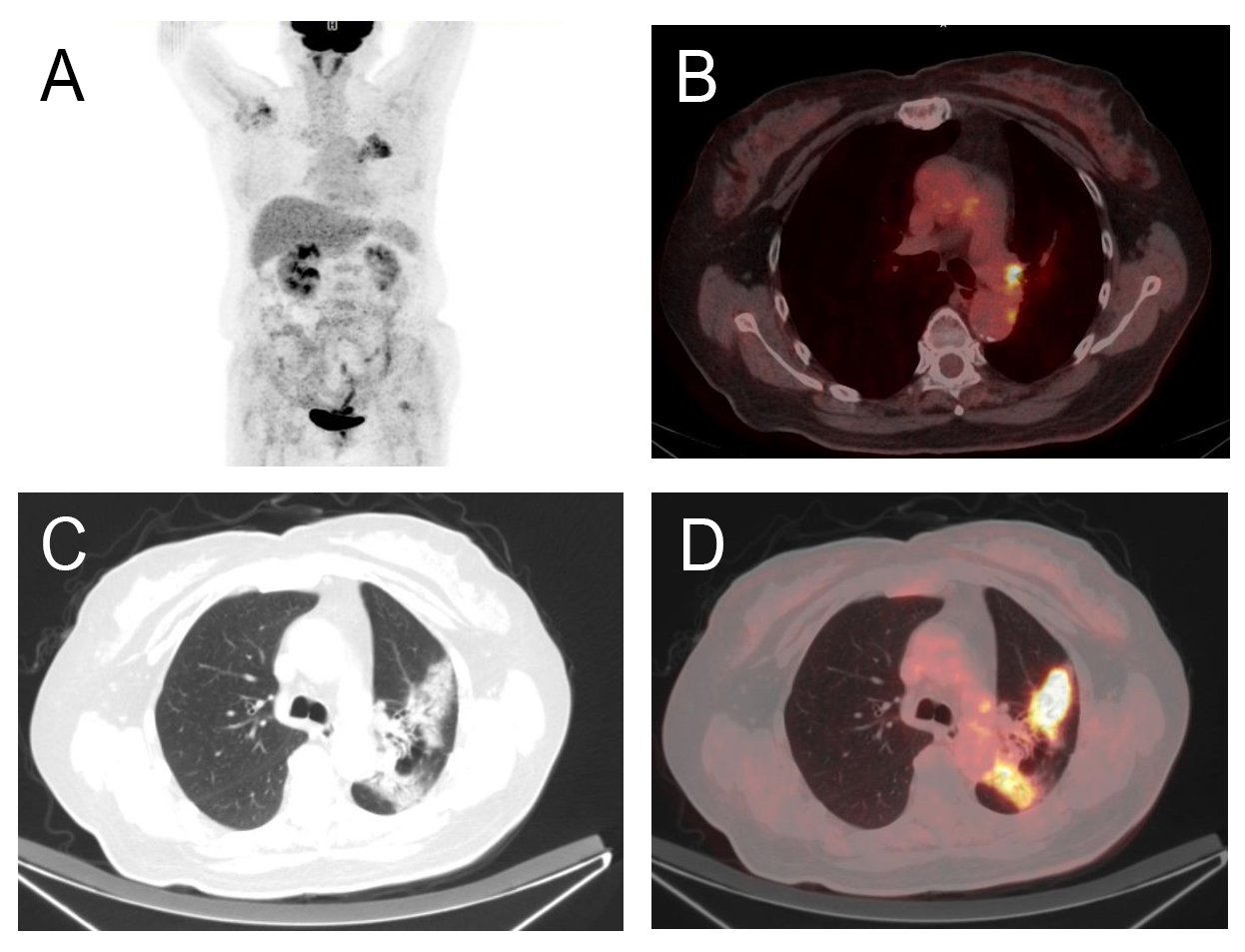
**

**Figure S1** Radiologic examination before surgery of Patient 3

PET/CT scan showed large patches of mixed ground-glass opacities at the apicoposterior segment of the upper lobe and posterior segment of the lower lobe of the left lung accompanied by the air bronchogram sign and cavity formation (C) with an SUVmax of 6.2 (D), enlarged lymph nodes in the left lung hilum and SUVmax of 4.8(B)
