# Supplementary material for: Neoadjuvant Programmed Cell Death Protein 1 Blockade Combined With Stereotactic Body Radiation Therapy for Stage III(N2) Non-Small Cell Lung Cancer: A Case Series
Source: Front Oncol. 2022 Mar 7;12:779251. doi: 10.3389/fonc.2022.779251 (PMC8936067; doi:10.3389/fonc.2022.779251)
Supplement: Supplementary file 2 [file Table_1.docx]

| Patient | History of smoking  (pack/years) | PS score | Pathologic type | EGFR and ALK test | Other gene mutations | TMB | PD-L1  22C3 | Radiologic evaluation | Time from last immunotherapy to surgery | Surgery procedure | Pathologic evaluation | Toxic side effects |
| --- | --- | --- | --- | --- | --- | --- | --- | --- | --- | --- | --- | --- |
| Patient 1 | 50 | 1 | Adenocarcinoma | EGFR  /ALK (−) | KRAS+  TP53+ | 18.46 Muts/Mb high | 70% | PR | 4 weeks | Thoracotomy surgery R0 | CPR | Grade 2 RF;  Grade 2 pruritus |
| Patient 2 | 40 | 1 | Adenocarcinoma | EGFR  ALK (−) | No | - | 0% | SD (shrunk by 29%) | 6 weeks | Thoracoscopy R0 | No-MPR  (20% residue) | No |
| Patient 3 | No | 1 | Adenocarcinoma | EGFR  ALK (−) | ERBB2+ | 9.3Muts/Mb | 10% | PR | 9 weeks | Thoracotomy surgery R0 | MPR | Postoperative chest pain |

Table S1. The patient and tumor characteristics.
